# A Dynamic Nomogram for 3-Month Prognosis for Acute Ischemic Stroke Patients After Endovascular Therapy: A Pooled Analysis in Southern China

**DOI:** 10.3389/fnagi.2021.796434

**Published:** 2021-12-13

**Authors:** Zhi-Xin Huang, Yong-Kun Li, Shi-Zhan Li, Xian-Jun Huang, Ying Chen, Quan-Long Hong, Qian-Kun Cai, Yun-Fei Han

**Affiliations:** ^1^Department of Neurology, Guangdong Second Provincial General Hospital, Guangzhou, China; ^2^Department of Neurology, The Second School of Clinical Medicine, Southern Medical University, Guangzhou, China; ^3^The School of Medicine, Jinan University, Guangzhou, China; ^4^Hengyang Medical School, University of South China, Hengyang, China; ^5^Department of Neurology, Fujian Provincial Hospital, Shengli Clinical College of Fujian Medical University, Fuzhou, China; ^6^Department of Neurology, Xiamen Key Laboratory of Brain Center, The First Affiliated Hospital of Xiamen University, Xiamen, China; ^7^Department of Neurology, The No. 1 People’s Hospital of Yulin, Yulin, China; ^8^Department of Neurology, Yijishan Hospital of Wannan Medical College, Wuhu, China; ^9^Department of Neurology, The First Hospital of Quanzhou Affiliated to Fujian Medical University, Quanzhou, China; ^10^Department of Neurology, The Second Affiliated Hospital of Fujian Medical University, Quanzhou, China; ^11^Department of Neurology, Medical School of Nanjing University, Jinling Hospital, Nanjing, China

**Keywords:** ischemic stroke, endovascular therapy, prognosis, nomogram, risk factor

## Abstract

Cerebral edema (CDE) is a common complication in patients with acute ischemic stroke (AIS) and can reduce the benefit of endovascular therapy (EVT). To determine whether certain risk factors are associated with a poor prognosis mediated by CDE after EVT. The 759 patients with anterior circulation stroke treated by EVT at three comprehensive stroke centers in China from January 2014 to October 2020 were analyzed. Patients underwent follow-up for 3 months after inclusion. The primary endpoint was a measure of a poor prognosis (modified Rankin Scale score ≥ 3) at 3 months assessed in all patients receiving EVT. Least absolute shrinkage and selection operator and multivariate logistic regression were used to select variables for the prognostic nomogram. Based on these variables, the nomogram was established and validated. In addition, structural equation modeling was used to explore the pathways linking CDE and a poor prognosis. Seven predictors were identified, namely, diabetes, age, baseline Alberta Stroke Program Early CT score, modified Thrombolysis in Cerebral Infarction score, early angiogenic CDE, National Institutes of Health Stroke Scale score, and collateral circulation. The nomogram consisting of these variables showed the best performance, with a large area under the curve in both the internal validation set (0.850; sensitivity, 0.737; specificity, 0.887) and external validation set (0.875; sensitivity, 0.752; specificity, 0.878). In addition, CDE (total path coefficient = 0.24, *P* < 0.001) served as a significant moderator. A nomogram for predicting a poor prognosis after EVT in AIS patients was established and validated with CDE as a moderator.

## Introduction

Endovascular therapy (EVT) has been recognized as the standard treatment for acute ischemic stroke (AIS) and is suitable for certain patients with anterior circulation infarction with large-vessel occlusion (Hill and Goyal, 2018). However, a significant proportion of AIS patients do not clinically improve despite timely EVT, successful revascularization of the occluded arteries, and reperfusion of the ischemic areas. Even after successful EVT, a high degree of variability in clinical outcomes remains among AIS patients; therefore, predicting functional outcomes may have important implications for the clinical management of these patients. The reasons for the lack of improvement in these cases are still not fully understood. Although five randomized EVT trials were performed recently to analyze factors affecting the 3-month prognosis (Goyal et al., 2016), most findings from these trials focused on the severity of stroke, site of occlusion, estimated time from symptom onset to treatment, and method of treatment, while few studies incorporated cerebral edema (CDE), especially in Chinese Han ethnics (Chen et al., 2019; Han et al., 2020). Most patients with AIS die from CDE, which can lead to reduced cerebral blood flow, increased intracranial pressure and neuronal death. Moreover, CDE is the second major pathomorphological feature of stroke, and it can be attributed to the focus of the infarct. In large cerebral infarctions, progressive ischemic edema can lead to serious complications and mass effects, with a mortality rate of up to 80% (Berrouschot et al., 1998), unless treated with early recanalization (Thoren et al., 2020). Thus, CDE may be a crucial modulator of clinical prognosis; However, the role of baseline patient characteristics and treatment modalities, among others, in modulating clinical prognosis during ischemic injury through the mediating effect of CDE has also not been established yet.

We hypothesized that certain risk factors are associated with a poor prognosis mediated by CDE in AIS patients after EVT. For example, other known risk factors for a poor prognosis after EVT include the stroke severity, as measured by the National Institutes of Health Stroke Scale (NIHSS), the recanalization status, and the baseline Alberta Stroke Program Early CT score (ASPECTS) (Yeo et al., 2016; Khan et al., 2017; Stracke et al., 2020). However, their impact on the prognosis of AIS after EVT remains uncertain; the predictive accuracy of individual variables is limited, and multivariate risk prediction tools based on demographic, clinical and neuroimaging characteristics may be more practical.

This study was performed to determine factors of a poor prognosis in patients with AIS despite the use of EVT to develop a dynamic nomogram for predicting a poor prognosis in such patients, and to explore the moderating role of CDE.

## Materials and Methods

### Participants

To ensure the generalizability of the study results, patients with anterior circulation infarction treated with EVT were pooled from 3 comprehensive stroke centers (Jinling Hospital from January 2014 to December 2018, Yijishan Hospital from July 2015 to October 2020 and the Second Affiliated Hospital of Fujian Medical University from January 2016 to December 2019). The uniform inclusion criteria were as follows: (1) age ≥ 18 years; (2) prestroke modified Rankin Scale (mRS) score < 2; and (3) occlusion of the internal carotid artery (ICA) or middle cerebral artery (MCA) (M1/M2) confirmed on preoperative imaging. The treatment options and methods have been previously described (Huang et al., 2021).

The collaborators collected datasets with the same clinical and imaging characteristics used in an external validation cohort from three other stroke centers, namely, the No.1 People’s Hospital of Yulin, Quanzhou First Hospital, and Shengli Clinical Medical College of Fujian Medical University, to validate the nomogram. Data were collected from a cohort of consecutive AIS patients admitted to these three hospitals from January 2017 to October 2020.

### Data Collection

All consecutive patients with anterior circulation AIS were prospectively documented. Baseline characteristics included demographics, vascular risk factors, medical history (diabetes mellitus, hypertension, and atrial fibrillation), Trial of ORG 10172 in Acute Stroke Treatment (TOAST) classification, stroke severity (measured by NIHSS), pre- and post-treatment imaging features, type of treatment, and complications.

The operational parameters included the estimated time from onset to puncture (OTP), time from stroke onset to reperfusion (OTR), location of occlusion, EVT approach (stent-first/aspiration-first/angioplasty-first), remedial treatment, and collateral circulation. Recanalization after EVT was evaluated by the interventional physician according to the modified Thrombolysis in Cerebral Infarction (mTICI) scoring system. Successful recanalization was defined as an mTICI score of 2b or 3. The collateral circulation was evaluated based on the retrograde contrast opacity of the vessels within the occluded region on delayed pretreatment digital subtraction angiography (DSA). The collateral circulation was scored as follows (Christoforidis et al., 2005) grade 0 if there was little or no significant reconstruction of the occluded vascular region or if the collaterals reached less than 1/3 of the occluded region; grade 1 if the collaterals reached less than 2/3 of the occluded region; and grade 2 if the side branches reached more than 2/3 of the region or the proximal main vessel. Neuroradiologists evaluated the severity of brain edema 24–72 h after EVT. Early angiogenic CDE was classified based on the published literature (Cheripelli et al., 2016), as follows: no swelling, 0 point; effacement of the lateral ventricle, 1 point; effacement of the lateral ventricle and third ventricle, 2 points; and midline shift, 3 points.

### Primary Outcome

The primary endpoint consisted of the functional outcome as assessed with the mRS in all patients 3 months after stroke. Good functional recovery was defined as an mRS score of 0–2, and poor functional recovery was defined as an mRS score ≥ 3.

### Statistical Analysis

R version 3.5.1, SPSS version 25.0 and AMOS 24.0 were used to perform the statistical analyses. Continuous variables are presented as the means ± standard deviation (SD) or as the median and interquartile range (IQR), and categorical variables are expressed as frequencies or percentages. First, to avoid case deletion due to missing baseline data in the multivariate analysis, a regression-switching approach was used to multiply impute the missing data (Supplementary Figure 1). The imputation model consisted of variables from the analytical model, imputed incomplete variables and complete variables that served as predictors. Second, to predict a poor prognosis in patients, the original data were randomly divided into two subsets, namely, the training set (70%) and the internal validation set (30%), in R. The data from the training set were analyzed using the least absolute shrinkage and selection operator (LASSO) regression method. LASSO regression is a method of data dimensional reduction that can be used to select predictors associated with the primary endpoint. Third, in the current study, patients were divided into good and poor prognosis groups based on neurological recovery (mRS score). Binary logistic regression was used to analyze the associations of factors with the 3-month prognosis. Moreover, a nomogram was constructed on the basis of the binary logistic regression analysis results to visualize the probability of a combination of factors affecting the prognosis. Fourth, Harrell’s concordance index (C-index) was used to quantify the performance of the nomograms. The imputed data were randomly divided into a training set (70%) and an internal validation set (30%) (Supplementary Figure 2). All data from the internal validation set were used for internal validation of the model. The nomogram was also externally validated using external validation sets from three other stroke centers. Discrimination and calibration were assessed using a bootstrapping method with 1,000 iterations. Additionally, decision curve analysis (DCA) was performed to independently evaluate the clinical value of the model based on the calculation of the patient’s net benefit at each threshold probability. The model was compared to the strategies of selecting all and selecting none; the strategy with the highest calculated net benefit was considered optimal, and the random forest algorithm was used to evaluate the importance of the predictors. Finally, an ordinal logistic regression analysis was performed to explore the factors influencing CDE, using CDE classification as the dependent variable and the related factors as independent variables. Then, structural equation modeling (SEM) was used to explore the pathways linking CDE and a poor prognosis. The structural impairments were assessed by the collateral status and the admission ASPECTS and NIHSS. Four parameters were used to evaluate model fitting: the root-mean-square-error of approximation (RMSEA), goodness-of-fit index (GFI), adjusted goodness-of-fit index (AGFI), and Akaike information criterion (AIC). Two-sided *P* < 0.05 was considered statistically significant.

## Results

This study involved 759 AIS patients, comprising 452 (59.6%) male participants and 307 (40.4%) female participants from three stroke centers in China. The mean age of all participants was 66.9 ± 11.5 years. Among the risk factors for stroke, hypertension was identified in 67.5% (512/759) of the patients, atrial fibrillation was identified in 48.2% (366/759), and diabetes was identified in 19.0% (144/759). The median time from onset to sheath insertion and the median duration of the operative procedure was 270 (IQR: 213–330) minutes and 73 (IQR: 50–109) minutes, respectively. Revascularization was achieved in 572 (75.4%) patients. Over the 3-month follow-up, 331 (43.6%) patients recovered well.

### Risk Factors and Risk Model Development

The patient dataset was randomly divided into a training set (*n* = 531 patients) and an internal validation set (*n* = 228 patients) (Table 1). There were 322 males (60.6%), 390 (73.4%) patients over 60 years old, and 228 with a good 3-month prognosis (42.9%) in the training set. The internal validation set, comprised 130 male patients (57.0%) and 98 female patients (43.0%). Within the validation set, 170 (74.6%) patients were older than 60 years, and 103 patients (45.2%) had a good 3-month prognosis. For external validation, the collaborators provided data only for the variables in the nomogram. The detailed demographic and clinical characteristics are shown in Table 1.

**TABLE 1:** Baseline clinical features and radiographic characteristics.

	Training set *n* = 531	Internal validation set *n* = 228	External validation set *n* = 219
**Demographics**			
Female, n (%)*	209 (39.4)	98 (43.0)	86 (39.3)
Age, years, mean (SD)*	67.1 (11.3)	66.5 (11.8)	64.7 (12.0)
**Medical history**			
Hypertension, n (%)	366 (68.9)	146 (64.0)	
Diabetes mellitus, n (%)*	105 (19.8)	39 (17.1)	51 (23.3)
Atrial fibrillation, n (%)	259 (48.8)	107 (46.9)	
**Clinical characteristics**			
Baseline SBP, mmHg, mean (SD)	147.1 (24.2)	145.0 (25.1)	
Baseline DBP, mmHg, mean (SD)	82.2 (13.8)	82.2 (16.2)	
Admission NIHSS, median (IQR)*	16 (13–20)	16 (12–19)	16 (13–20)
Baseline ASPECTS, n (%)*			
<8	126 (23.7)	60 (26.3)	38 (17.4)
≥8	405 (76.3)	168 (73.7)	181 (82.6)
OTP, min, median (IQR)	270 (210–330)	270.0 (215.8–332.3)	
OTR, min, median (IQR)	349 (286–445)	360 (286–420)	
Collateral status, n (%)*			
Grade 0	105 (19.8)	51 (22.4)	64 (29.2)
Grade 1	192 (36.2)	84 (36.8)	99 (45.2)
Grade 2	234 (44.1)	93 (40.8)	56 (25.6)
mTICI score > 2b, n (%)	404 (76.1)	169 (74.1)	185 (84.5)
First treatment, n (%)			
Stent retriever	399 (75.1)	165 (72.4)	
Contact aspiration	77 (14.5)	35 (15.4)	
Angioplasty	55 (10.4)	28 (12.3)	
Tandem, n (%)	67 (12.6)	27 (11.8)	
Occlusion site, n (%)			
ICA	224 (42.2)	107 (46.9)	
MCA (M1)	269 (50.7)	106 (46.5)	
M2 and beyond	38 (7.2)	15 (6.6)	
CDE*			
0 point	357 (67.2)	156 (68.4)	169 (77.2)
1 point	51 (9.6)	20 (8.8)	5 (2.3)
2 points	6 (1.1)	2 (0.9)	2 (0.9)
3 points	117 (22.0)	50 (21.9)	43 (19.6)
Remedial treatment, n (%)	89 (16.8)	50 (21.9)	
TOAST classification, n (%)			
Atherosclerotic	173 (32.6)	75 (32.9)	
Cardioembolic	299 (56.3)	126 (55.3)	
Others	59 (11.1)	27 (11.8)	

*For the external validation set, only those variables used in the nomogram were included.

**FIGURE 1:**
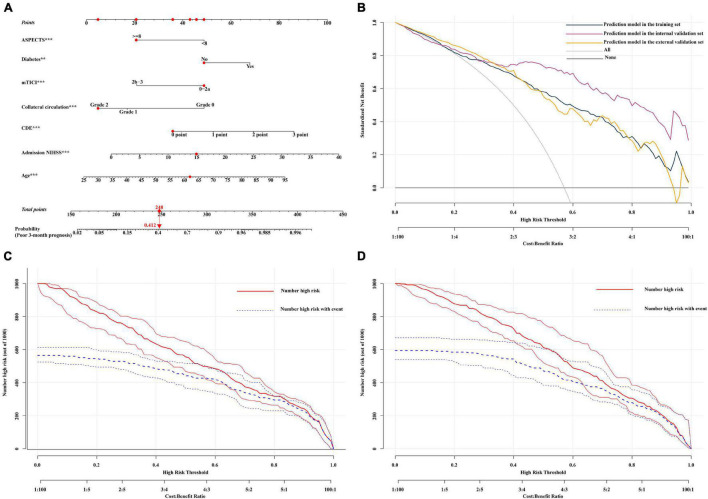
**(A)** Nomogram for the prediction of a poor 3-month prognosis. Scores were based on the ASPECTS, age, mTICI score, diabetes, CDE, NIHSS, and collateral circulation status by drawing a line from the corresponding value upward to the “score line.” The “total score” was calculated as the sum of the individual scores for each of the 7 variables included in the nomogram. For example, we will consider a 62-year-old diabetic patient had an mTICI score of 0-2a with a CDE grade of 0 points, a collateral circulation grade of 2, an NIHSS of 15, and an ASPECTS of ≥ 8. Thus, the sum of the total risk points was 248, and a vertical line can be drawn down to the “3-month poor prognosis probability” axis. For this patient, the prognostic probability was 41.2%. **(B)** Decision curve analysis (DCA) of the nomogram in the validation set. The *x*-axis shows the threshold probability. The *y*-axis shows the net benefit. The gray line shows the net benefit of the treatment strategy for all patients. The black line shows the net benefit of the strategy for patients without treatment. The yellow, dark green, and purple lines represent the nomogram. This nomogram was used to assess the probability of a poor prognosis in a specific patient with AIS treated with EVT. Patients with a higher risk of a poor prognosis after EVT may require further treatment, such as decompressive craniectomy, while patients with a lower risk of mortality may not require further treatment. The distinction between patients with a high and low risk of mortality is the main purpose of this figure. In this study, the reference risk was calculated by assuming that all patients required further treatment to prevent adverse events, whereas zero net benefit was defined as no patients requiring further treatment. The threshold probability is defined at the point where the expected benefit of further treatment equals the expected benefit of avoiding further treatment. For any given probability threshold, the nomogram with the largest net benefit would be the most ideal model (**(C)** for internal validation and **(D)** for external validation). The clinical impact curve of the nomogram of a high risk of a poor prognosis, in which the predicted high-risk probability coincides well with the actual high-risk probability and has a superior standardized net benefit, including 95% confidence intervals (CIs). NIHSS, National Institutes of Health Stroke Scale; CDE, early angiogenic CDE score.

After the selection of variables through LASSO regression models, age, ASPECTS, mTICI score, sex, remedial treatment, hypertension, diabetes, atrial fibrillation, location of occlusion, CDE, NIHSS, and collateral circulation were identified as the best subset of risk factors to include in the model for predicting a poor prognosis (Supplementary Figure 3 and Supplementary Table 1). Figure 1A shows the multiple logistic regression analysis for a poor prognosis within 3 months (the higher the total score, based on the sum of points assigned to each predictor in the nomogram, the higher the risk of a poor prognosis). In addition, to make the model more accessible to clinicians, a dynamic web-based version of the nomogram was created. The interface of this web version is shown in Supplementary Figure 4. On the right side of the interface, the 3-month prognostic probability and 95% confidence interval (CI) can be obtained by entering the appropriate patient data and then clicking the button at the bottom of the page. The predicted probabilities of the online tool are consistent with those of the static nomogram.^1^

**FIGURE 2:**
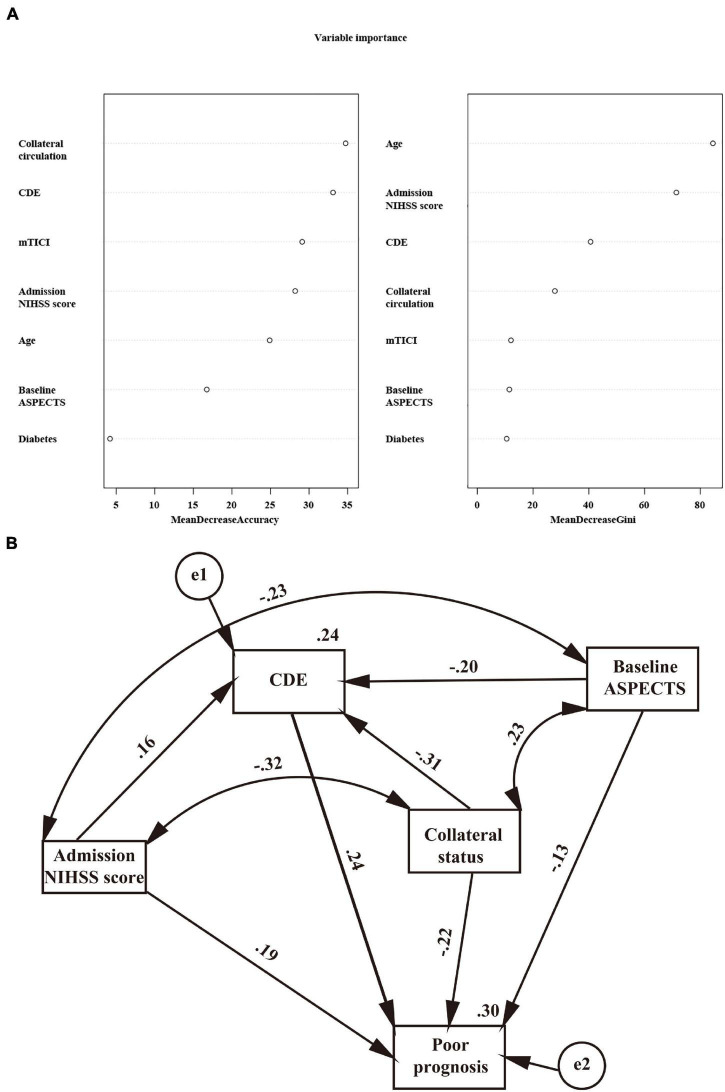
**(A)** Importance values of clinical variables. MeanDecreaseAccuracy indicates a decrease in accuracy after variable substitution; MeanDecreaseGini indicates a decrease in the Gini coefficient after variable substitution. A higher value indicates that the variable is more important. **(B)** Regressions from the structural equation model on the relationship of the admission NIHSS, baseline ASPECTS, collateral status, and CDE with a poor prognosis. Standardized coefficients are presented; all *P* < 0.001.

DCA can be used to estimate the net benefit of a model based on the difference between true-positive and false-positive results and is widely used to assess whether nomogram-assisted decision-making improves patient outcomes. DCA showed that the nomogram could be easily applied and used to make valuable and useful judgments (Figure 1B). The red curve (number at high risk) represents the number of people classified as positive (high risk) by the model at each threshold probability; the blue curve (number at high risk with the outcome) represents the number of true positives at each threshold probability (Figures 1C,D). The C-index (95% CI) and area under the receiver operating characteristic (ROC) curve (AUC) for the model was 0.903 (0.883–0.924) and 0.903 in the internal validation set and 0.831 (0.804–0.858) and 0.831 in the external validation set, respectively. In addition, the nomogram model performed well, with a high AUC in both the internal validation set (sensitivity, 0.856; specificity, 0.874) and external validation set (sensitivity, 0.615; specificity, 0.888) (Supplementary Figures 5A,B). According to the calibration plot, the mean absolute error for a poor 3-month prognosis in the internal and external validation sets were 0.018 and 0.029, respectively (Supplementary Figures 5C,D). Among the included variables, statistically significant prognostic predictors were severe neurological deficits [odds ratio (OR), 1.092; 95% CI, 1.050–1.135], age (OR, 1.049; 95% CI, 1.031–1.067), diabetes (OR, 2.087; 95% CI, 1.286–3.386), CDE (OR, 1.925; 95% CI, 1.550–2.390), collateral circulation (OR, 0.476; 95% CI, 0.363–0.625), ASPECTS (OR, 0.329; 95% CI, 0.200–0.541), and recanalization status (OR, 0.338; 95% CI, 0.210–0.546). A variable importance measure was used to evaluate the impact of each variable. The order of importance of the variables was as follows (highest to lowest): age, NIHSS, CDE, collateral circulation, mTICI score, ASPECTS, and diabetes (Figure 2A).

### Path Analysis of the Effects of Cerebral Edema on Poor Prognosis

After adjusting for confounders in the ordinal regression model, it was found that patients with higher diastolic blood pressure (DBP) (OR = 1.012, 95% CI: 1.001–1.022, *P* = 0.028), severe neurological deficits (OR = 1.053, 95% CI: 1.029–1.078, *P* < 0.001), a lower ASPECTS (OR = 1.997, 95% CI: 1.528–2.611, *P* < 0.001) and a poor collateral circulation had more severe CDE (Table 2). The significant linking pathways from CDE to a poor prognosis in the SEM model are displayed in Figure 2B. In this SEM model, collateral status and the admission ASPECTS and NIHSS were not only directly (standardized beta estimates −0.22, −0.13, and 0.19, respectively, all *P* < 0.001) but also indirectly linked with a poor prognosis. CDE (total path coefficient = 0.24, *P* < 0.001) served as a significant mediator.

**TABLE 2:** Ordered logistic regression analysis of factors affecting the grading of brain edema after treatment in patients with acute ischemic stroke.

Factors	OR (95% CI)	*P*-value
Age, years	0.990 (0.977–1.003)	0.135
Sex		
Male	0.964 (0.727–1.280)	0.800
Female	Ref.	
Baseline SBP, mmHg	1.000 (0.994–1.006)	0.985
Baseline DBP, mmHg	1.012 (1.001–1.022)	0.028
Admission NIHSS	1.053 (1.029–1.078)	<0.001
Hypertension		
No	0.730 (0.537–0.994)	0.046
Yes	Ref.	
Diabetes mellitus		
No	0.793 (0.579–1.086)	0.148
Yes	Ref.	
Atrial fibrillation		
No	1.436 (0.998–2.067)	0.051
Yes	Ref.	
Tandem		
No	0.966 (0.620–1.506)	0.879
Yes	Ref.	
Baseline ASPECTS		
<8	1.997 (1.528–2.611)	<0.001
≥8	Ref.	
Collateral status		
Grade 0	4.126 (2.819–6.040)	<0.001
Grade 1	2.497 (1.742–3.580)	<0.001
Grade 2	Ref.	
TOAST classification		
Atherosclerotic	0.754 (0.474–1.201)	0.235
Cardioembolic	1.081 (0.645–1.812)	0.767
Others	Ref.	
Occlusion site		
ICA	3.249 (1.601–6.592)	0.001
MCA (M1)	1.496 (0.730–3.064)	0.271
M2 and beyond	Ref.	

## Discussion

The main finding of our study is that a poor prognosis in AIS patients with anterior circulation occlusion was associated with the following clinical and imaging determinants: age, diabetes, collateral circulation, CDE, NIHSS, ASPECTS at baseline, and mTICI score. After random forest model training, the variables were, in descending order of importance, age, NIHSS, CDE, collateral circulation, mTICI score, ASPECTS, and diabetes. In the present study, CDE served as an important mediator of a poor prognosis in AIS patients.

We found that older patients had significantly higher rates of a poor 3-month prognosis than younger patients. We also found that the presence of diabetes, a higher NIHSS on admission and a poor collateral circulation were independent predictors of a poor 3-month prognosis. First, many studies have examined the results of EVT in older adults (Lima et al., 2016; Sallustio et al., 2017). Overall, our findings are consistent with the findings of these studies, with higher rates of a poor prognosis in the elderly population. Advanced age is well known to be associated with insufficient collateral circulation in multiple tissues, increasing the severity of ischemic injury (Faber et al., 2011). Second, a poor pretreatment collateral circulation may reduce the rates of recanalization and reperfusion in EVT for AIS (Leng et al., 2016). If the collateral circulation is poor and the ischemic core lesion is located in a critical brain region, such as the motor areas, patients are usually left with severe disability even after early reperfusion. These patients may present with severe clinical symptoms hours after symptom onset but experience complete neurological recovery after reperfusion (Yassi et al., 2015). Third, the angiographic collateral score (mTICI score) obtained during EVT is used to predict which patients are most likely to achieve successful revascularization and is associated with increased infarction and the clinical outcome (Marks et al., 2014). Fourth, previous studies have suggested that despite successful revascularization, a high NIHSS in patients is associated with a poor postoperative prognosis (Linfante et al., 2016) because higher NIHSS are associated with increased rates of ineffective recanalization and hemorrhagic transformation (Hussein et al., 2010; Kuntze Soderqvist et al., 2014).

To our knowledge, few studies have been conducted to investigate the relationship of ischemic edema with functional outcomes using CDE as a simple and practical metric in AIS patients after EVT. These findings may have important clinical implications in several areas, including patient selection for interventions, prognostic evaluation, and decision-making regarding adjuvant regimens with antiedematous agents in patients with a poor collateral circulation. First, the influence of the collateral circulation on the prognosis of ischemic stroke is well established (Vagal et al., 2018). The influence of a poor collateral circulation on the clinical prognosis may be due not only to increased lesion growth but also to an increase in ischemic CDE (Galego et al., 2018). Significant CDE with progressive tissue fluid uptake may lead to increased interstitial pressure after the occlusion of a large vessel. This may lead to increased resistance of collateral arterioles and downstream perforating arterioles in the low-perfusion area after cerebral infarction. Subsequently, ischemic edema may worsen, leading to a poor prognosis even after successful EVT. Thus, patients with minimal or no early brain edema may maintain an adequate collateral circulation and may be relatively likely to benefit from EVT. However, patients with an initially poor collateral circulation may rapidly progress to severe CDE. In these patients, the impact of the formation of pronounced early edema on clinical outcomes may be more time-sensitive (Marks et al., 2014). In conclusion, CDE may serve as a predictor of functional outcomes. Second, the development of edema is the result of multiple factors; persistent occlusion is an important cause, as are the size of the ischemic lesion, the collateral circulation status, and the size of the occluded artery, as reflected by the NIHSS and the size of the infarct core. Third, at the tissue level, edema is caused by a gradual increase in blood-brain barrier permeability and disruption (Paciaroni et al., 2012; Horsch et al., 2016; Fredriksson et al., 2017; Shi et al., 2018). Ischemia and hypoxia cause damage to brain tissue, followed by the depletion and failure of ATP-dependent ion pumps, leading to the dysfunction of neuronal electrical function and energy metabolism (Kulik et al., 2008). In addition, cerebral capillary dysfunction caused by ischemia, hypoxia, and reperfusion leads to the progressive deterioration of blood-brain barrier permeability, resulting in cytotoxic edema, ionic edema, vasogenic edema, and hemorrhagic transformation (Simard et al., 2007). In summary, the development and progression of CDE must be closely monitored and controlled after EVT in AIS patients.

Our research has certain limitations. First, this was a retrospective analysis of a small sample of data from three centers. Hence, our results need to be validated in a large-scale prospective multicenter study. Second, CDE was assessed by measuring CDE, which was based on a morphometric analysis and an indirect measure of the mass effect, rather than a direct calculation of the fluid content. Third, there was a lack of external validation across different ethnicities and a relatively small sample size in the validation model. Therefore, future multicenter prospective studies including participants of different ethnicities and larger sample sizes should be conducted to validate the accuracy of the model. Fourth, measuring CDE at 24 h may not capture the maximum values of CDE which generally peaks between day 2–4 after stroke, especially in patients with persistent arterial occlusion who may have been suffering infarct growth beyond 24-h. At the same time, different treatments such as mannitol may influence edema.

Nevertheless, one advantage of the present study is the relatively large cohort of AIS patients treated with EVT from multicenter; compared with nomograms, dynamic nomograms often provide more tailored risk predictions that facilitate management-related decisions. Importantly, the overall predictive performance and clinical utility of our model were also well validated in external cohorts. In addition, we created a prognostic dynamic nomogram in an online webserver. Without the need to install specific software, clinicians can access can access nomogram calculators online anywhere anytime. This will undoubtedly simplify the application and facilitate its use in clinical practice. Also, this paper provides a new insight to clinicians for predicting the probability of 3-month prognosis in AIS patients undergoing EVT, supporting therapeutic decision in this clinical setting.

## Conclusion

In conclusion, a newly developed nomogram for predicting a poor prognosis at 3 months showed that an older age, diabetes, a higher NIHSS, a poor collateral circulation, a lower ASPECTS at baseline, a lower mTICI score, and in particular, a higher level of CDE were associated with a poor prognosis in AIS patients eligible for EVT. Remarkably, for the first time to our knowledge, CDE was also found to be an important mediator of a poor prognosis in AIS patients. Future multicenter prospective studies are needed to validate the findings in different ethnic groups.

## Data Availability Statement

The raw data supporting the conclusions of this article will be made available by the authors, without undue reservation.

## Ethics Statement

This retrospective study was approved by the local ethics committee and conducted in accordance with the relevant guidelines. The requirement for informed consent was waived. Written informed consent for participation was not required for this study in accordance with the national legislation and the institutional requirements.

## Author Contributions

Z-XH and X-JH: guarantors of integrity of entire study. Z-XH, X-JH, Y-KL, and S-ZL: literature research. Z-XH, X-JH, Y-KL, S-ZL, Q-LH, Q-KC, and Y-FH: clinical studies. Z-XH and YC: statistical analysis. Z-XH, X-JH, Y-KL, and S-ZL: manuscript editing. All authors: study concepts and study design or data acquisition or data analysis and interpretation, manuscript drafting or manuscript revision for important intellectual content, approval of final version of submitted manuscript, and agreed to ensure any questions related to the work are appropriately resolved.

## Conflict of Interest

The authors declare that the research was conducted in the absence of any commercial or financial relationships that could be construed as a potential conflict of interest.

## Publisher’s Note

All claims expressed in this article are solely those of the authors and do not necessarily represent those of their affiliated organizations, or those of the publisher, the editors and the reviewers. Any product that may be evaluated in this article, or claim that may be made by its manufacturer, is not guaranteed or endorsed by the publisher.

## Footnotes

1
https://odywong.shinyapps.io/PredictingpPrognosis/
