# Supplementary material for: A Dynamic Nomogram for 3-Month Prognosis for Acute Ischemic Stroke Patients After Endovascular Therapy: A Pooled Analysis in Southern China
Source: Front Aging Neurosci. 2021 Dec 13;13:796434. doi: 10.3389/fnagi.2021.796434 (PMC8710662; doi:10.3389/fnagi.2021.796434)
Supplement: Supplementary file 1 [file Data_Sheet_1.PDF]

SUPPLEMENTAL MATERIAL

Title:
A Dynamic Nomogram for 3-Month Prognosis for Acute Ischemic Stroke Patients after Endovascular Therapy: A Pooled Analysis in Southern China

Cover title
Prognostic nomogram for AIS

Number of Supplementary Tables: 1
Number of Supplementary Figures: 5

Table S1. Comparison of demographic and therapeutic characteristics of the two study groups (Poor prognosis)

**Parameters**	**Three-month adverse prognosis (mRS ≥3)**		
	**Yes (n=428)**	**No (n=331)**	*P* value
Demographics			
Female, n (%)	194 (45.3)	113 (34.1)	0.002
Age, years, mean (SD)	69.4 (11.0)	63.7 (11.3)	<0.001
Medical history			
Hypertension, n (%)	306 (71.5)	206 (62.2)	0.007
Diabetes mellitus, n (%)	100 (23.4)	44 (13.3)	<0.001
Atrial fibrillation, n (%)	243 (56.8)	123 (37.2)	<0.001
Clinical characteristics			
Baseline SBP, mmHg, mean (SD)	148.8 (25.5)	143.7 (22.7)	0.005
Missing, n (%)	11 (2.6)	10 (3.0)	
Baseline DBP, mmHg, mean (SD)	82.9 (15.4)	81.3 (13.3)	0.135
Missing, n (%)	11 (2.6)	10 (3.0)	
Admission NIHSS score, median (IQR)	18 (14-21)	14 (11-17)	<0.001
Baseline ASPECTS, median (IQR)	8 (7-9)	9 (8-10)	<0.001

Missing, n (%)	3 (0.7)	0 (0)	
OTP, min, median (IQR)	266.0 (210.0-330.0)	278.5 (218.5-335.3)	0.405
Missing, n (%)	0 (0)	1 (0.3)	
OTR, min, median (IQR)	355.0 (290.0-446.0)	350.0 (279.5-425.5)	0.256
Missing, n (%)	3 (0.7)	2 (0.6)	
Collateral status, n (%)			<0.001
grade 0	139 (32.5)	17 (5.1)	
grade 1	171 (40.0)	105 (31.7)	
grade 2	118 (27.6)	209 (63.1)	
mTICI>2b, No. (%)	280 (65.4)	292 (88.2)	<0.001
Missing, n (%)	1 (0.2)	0 (0)	
First treatment, No. (%)			0.003
Stent retriever	328 (76.6)	232 (70.1)	
Contact aspiration	65 (15.2)	47 (14.2)	
Angioplasty	32 (7.5)	51 (15.4)	
Missing, n (%)	3 (0.7)	1 (0.3)	
Tandem, No. (%)	43 (10.3)	49 (14.8)	0.042

Missing, n (%)	10 (2.3)	10 (3.0)	
Occlusion site, No. (%)			<0.001
ICA	227 (53.0)	104 (31.4)	
MCA (M1)	174 (40.7)	201 (60.7)	
M2 and beyond	27 (6.3)	26 (7.9)	
CDE			<0.001
grade 1	216 (50.5)	297 (89.7)	
grade 2	50 (11.7)	21 (6.3)	
grade 3	8 (1.9)	0 (0)	
grade 4	154 (36.0)	13 (3.9)	
Remedial treatment, No. (%)	93 (21.9)	45 (13.6)	0.004
Missing, n (%)	3(0.7)	1 (0.3)	
TOAST classification, No. (%)			<0.001
Atherosclerotic	114 (26.6)	134 (40.5)	
Cardioembolic	272 (63.6)	153 (46.2)	
Others	42 (9.8)	44 (13.3)	

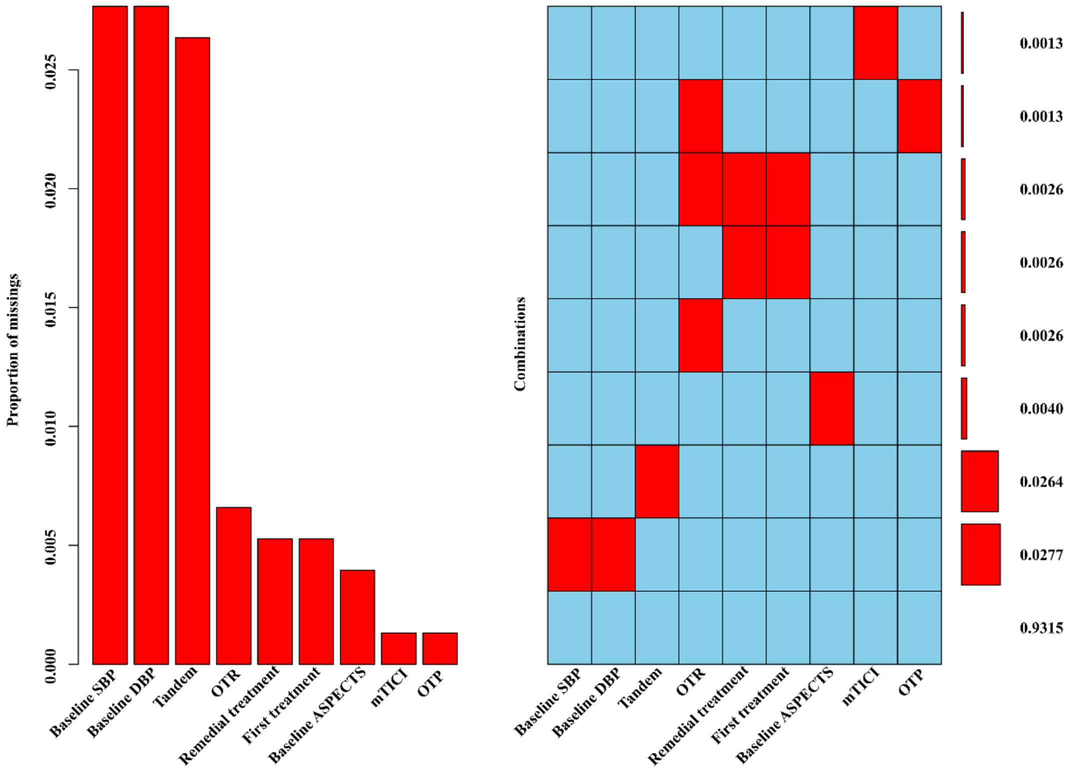

Figure S1. Missingness patterns in the data from the present study. (left) Proportion of total missingness; (right) proportion of each missingness pattern. Red, missing values.

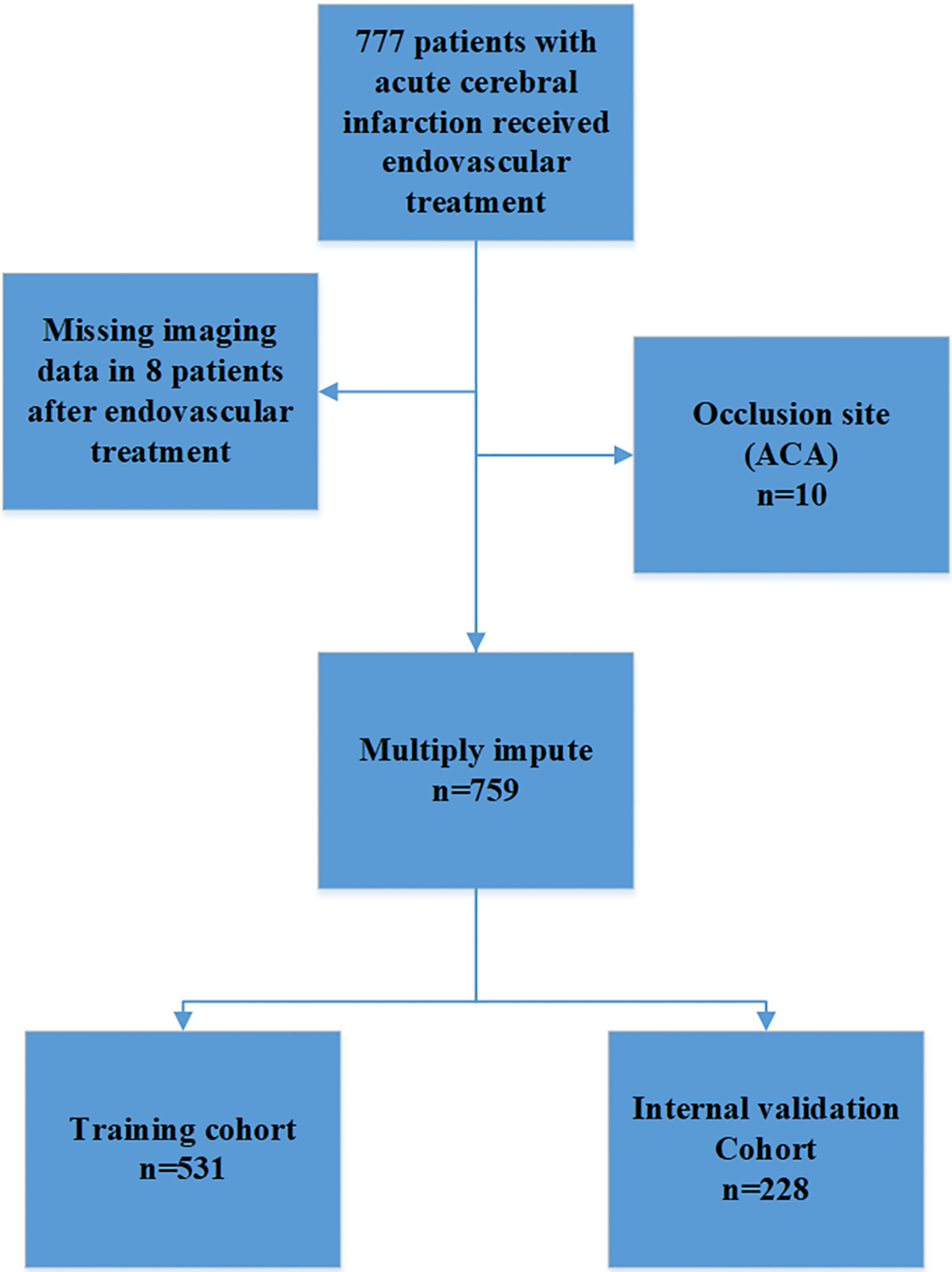

Figure S2. Flow chart showing study participant selection.

A

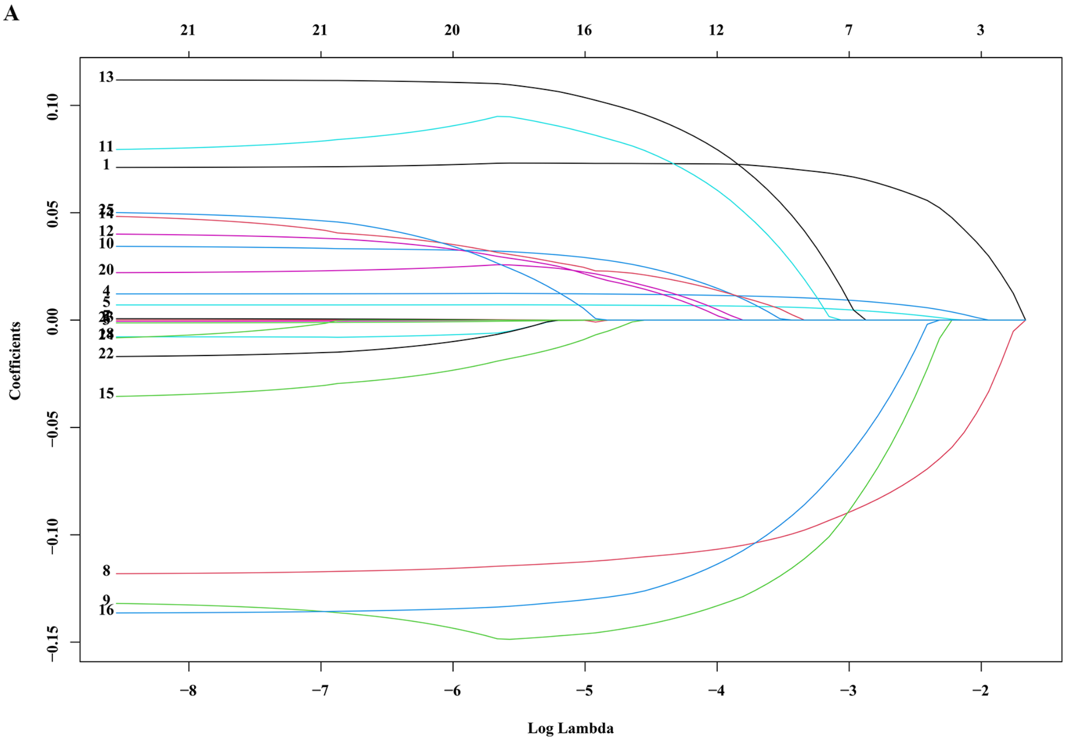

B

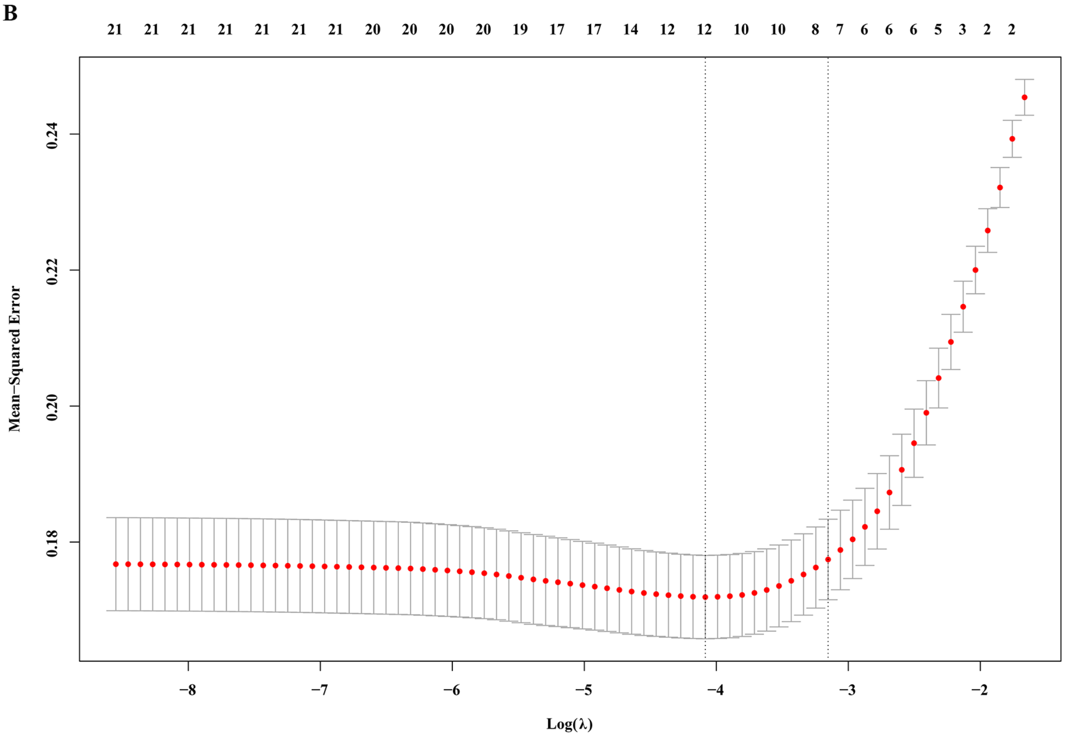

Figure S3. Predictor selection using the LASSO logistic regression model. (A) LASSO coefficient profiles of the 19 clinical features. (B) The optimal penalty coefficient lambda (λ) in the LASSO model was determined using 16-fold cross-validation and the minimum criterion. The dashed vertical line was plotted over the value selected using 10-fold cross-validation in Figure B, for which the optimal λ led to 12 nonzero coefficients.

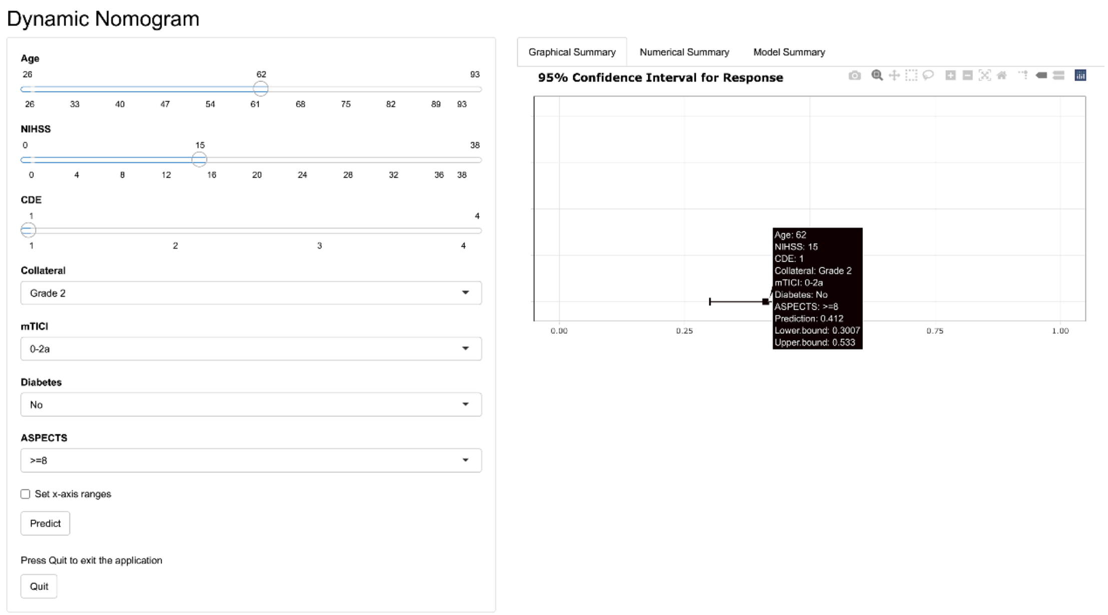

Figure S4. The dynamic nomogram of 3-months poor prognosis.

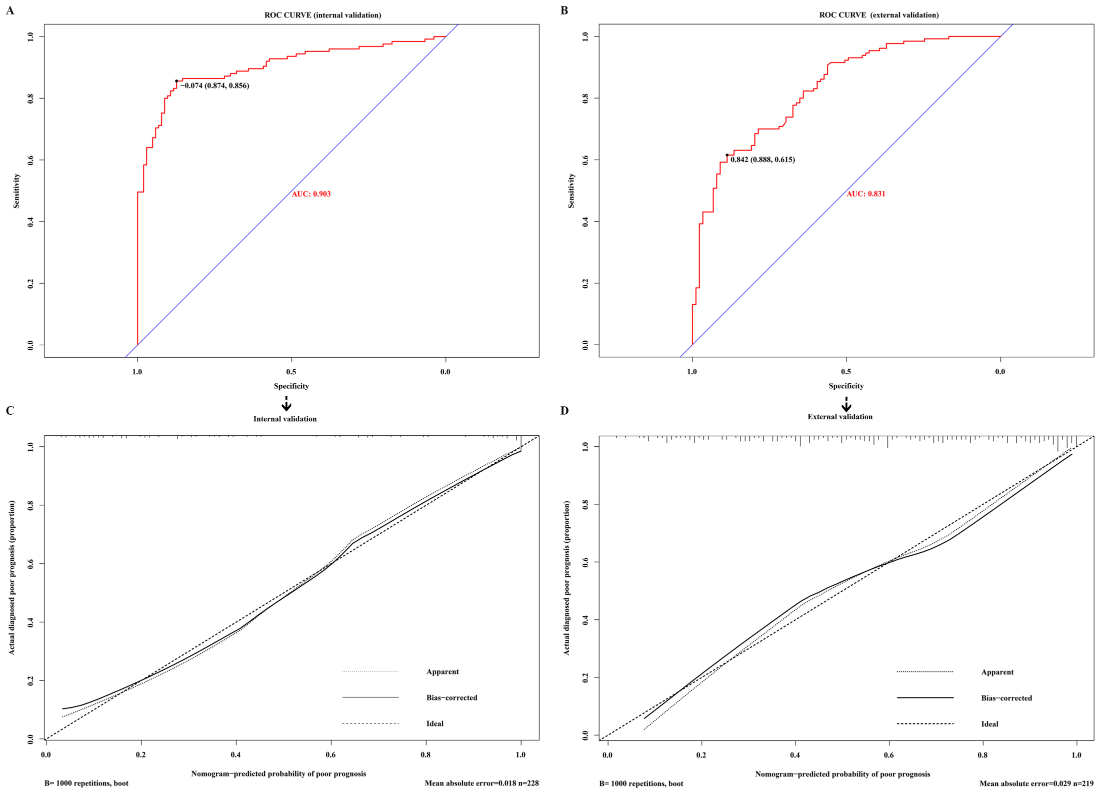

Figure S5 (A and B) The receiver operating characteristic curve for the discriminatory ability of the nomogram. The nomogram showed a strong discriminatory ability, with an area under the curve of 0.903, a specificity of 0.874, and a sensitivity of 0.856 for the internal validation set and corresponding values of 0.831, 0.888 and 0.615 for the external validation set. **(C and D)** The nomogram model calibration curve in the internal and external validation sets. The y-axis shows the actual probability, and the x-axis shows the predicted probability. The dashed line represents the reference line where an ideal nomogram would lie. The dotted line represents the performance of the nomogram, whereas the solid line corrects any bias in the nomogram.
